# Advances in Photocatalytic Membrane Reactor

**DOI:** 10.3390/membranes13060541

**Published:** 2023-05-23

**Authors:** Julie Mendret, Stephan Brosillon

**Affiliations:** IEM (Institut Européen des Membranes), UMR 5635 (CNRS-ENSCM-UM2), Université de Montpellier, 34095 Montpellier, France

Photocatalytic membrane reactors (PMRs) are a promising technology that combines the benefits of photocatalysis and membrane separation. These reactors are used in a variety of applications, including water and wastewater treatment, air purification, and organic synthesis. The Special Issue “Advances in Photocatalytic Membrane Reactors” in *Membranes* presents the latest research and developments in this field.

The Special Issue includes six papers, each of which covers different aspects of PMRs. The first paper focuses on addressing the issue of indoor air pollution caused by reactive ammonia (NH_3_) [1]. The researchers developed hollow fiber membranes based on Al_2_O_3_ and functionalized them with nitrogen-doped titanium dioxide (N-TiO_2_). They used a dip-coating technique to deposit thin films of TiO_2_ and N-TiO_2_ onto the Al_2_O_3_ membranes. The effectiveness of these membranes for photocatalytic degradation of NH_3_ gas was tested using both UV and visible light irradiation at room temperature. The study examined the impact of nitrogen doping on the photocatalytic capacity of TiO_2_ and explored how the performance of a prototype lab-scale photocatalytic membrane reactor, with varying numbers of membranes, was affected under different light conditions. Notably, the results demonstrated that the N-TiO_2_ photocatalyst, when combined with the Al_2_O_3_-based hollow fiber membranes, efficiently reduced the concentration of gaseous NH_3_ to zero within just fifteen minutes under UV and visible light. Increasing the number of functionalized membranes further enhanced the capacity for NH_3_ degradation.

In a second paper, Roubaud et al., examine the stability of PVDF-PVP-TiO_2_ hollow fiber membranes under UV irradiation to design a photocatalytic self-cleaning/low-fouling membrane [2]. The researchers investigated the impact of irradiation power, aqueous environment composition, and fouling state on membrane properties. Through SEM observations, chemical analysis, and tensile strength measurements, they found that pristine membranes exposed to UV irradiation in ultra-pure water experienced significant degradation due to OH° radicals. However, when methylene blue, a model pollutant, was present in the water, the radicals preferentially reacted with it rather than the membranes, preserving the membrane’s original properties. The presence of a pre-fouling adsorbed BSA layer on the membrane surface delayed membrane aging, as the BSA layer acted as a sacrificial target for the radicals instead of the membrane material. The degradation of the BSA layer further confirmed the self-cleaning properties of the membrane. However, when the membranes were pre-fouled by filtration of a BSA solution, aging delay was less effective because the trapped BSA molecules within the membrane pores shielded them from direct radical reactions, leading to increased degradation of the membrane material.

The third paper investigates the use of BiOCl-based nanocomposites as photocatalytic membranes for water desalination in reverse osmosis systems [3]. A molecular dynamic simulation was employed to design the molecular structure of BiOCl, BiOCl/Ag_2_S, and BiOCl/Bi_2_O_3_ heterojunctions, and their electronic properties, mechanical properties, and water desalination performance were evaluated. Geometry optimization and simulation tools were used for optimization and analysis. The nanocomposites demonstrated enhanced electronic and mechanical properties compared to materials like graphene and MoS_2_, and they exhibited improved salt rejection and water permeability. BiOCl and BiOCl/Ag_2_S exhibited an ideal bandgap for semiconductor photocatalysts, with a salt rejection of 98% achieved at 10 MPa pressure. BiOCl/Bi_2_O_3_ showed higher salt rejection, while BiOCl/Ag_2_S had higher water permeability. The mechanical stability of BiOCl/Ag_2_S and BiOCl/Bi_2_O_3_ surpassed that of MoS_2_. Overall, BiOCl/Ag_2_S was identified as the most suitable photocatalytic nanocomposite membrane among the three.

In the fourth paper, Tran et al. examined the anti-fouling performance of PVDF-TiO_2_ composite membranes by assessing their permeate flux with different types of synthetic feed solutions [4]. The membranes were subjected to photo-filtration, involving continuous UV irradiation, using solutions containing inorganic and organic components commonly found in drinking/natural water. The findings revealed that inorganic fouling was unlikely to occur on PVDF-TiO_2_ membranes, and the presence of typical inorganic ions in drinking water did not affect their performance. However, when a small amount of Cu^2+^ coexisted with HCO_3_^−^ in the feed solution, inorganic fouling occurred, leading to a significant decline in flux and hindering the photo-induced properties of the membranes. Regarding organic fouling solutions, the membranes demonstrated a strong resistance to sodium alginate fouling and relatively less resistance to humic acids. The membranes did not exhibit superior separation efficiency when operated in photo-filtration mode, as the rejection rates of the foulants under photo-filtration were not higher compared to normal filtration. The photodegradation of humic substances into smaller compounds during photo-filtration resulted in a lower rejection rate. Nonetheless, photo-filtration of these organic foulants still offered a higher permeate flux than normal filtration up to certain concentration thresholds (5 mg/L for humic acids and 50 mg/L for sodium alginate).

The fifth paper aimed to investigate the influence of preparation parameters, specifically the preparation temperature, on the structure, properties, and performance of PVDF-TiO_2_ composite membranes [5]. The membranes were fabricated using the non-solvent-induced phase separation (NIPS) method with a TiO_2_ content of 20 wt% in PVDF. A systematic approach was employed to understand the evolution of membrane formation and properties as the preparation temperature varied. The resulting membranes exhibited a high porosity and significantly improved permeate flux compared to neat PVDF membranes, but their mechanical strength was reduced. Notably, an increase in preparation temperature led to a significant transition in membrane morphology, characterized by a gradual reduction in finger-like macrovoids. Various membrane properties, including permeability, porosity, thermal and mechanical properties, and compression behavior, were also influenced accordingly. The study utilized ternary phase diagrams, examined solvent–nonsolvent exchange rates, and directly observed membrane formation during phase separation, providing insights into the observed evolution of membrane properties.

The last paper is a comprehensive review which discusses the discrepancies in traditional kinetic models, fluid flow dynamics, radiation emission and absorption, and their impact on upscaling and reactor design [6]. Computational and analytical approaches to develop high-throughput, efficient, and energy-effective PMR systems are provided, considering catalysts, fluid dynamics, thickness, geometry, and light sources. Two main PMR types are thoroughly described, and influential factors for PMRs are discussed to guide the development of an ideal reactor. Additionally, the application of PMRs is evaluated not only for removing endocrine-disrupting compounds (EDCs) but also for dye, oil, heavy metal, and pesticide removal from wastewater.

Overall, this Special Issue provides a comprehensive overview of the latest developments in the field of photocatalytic membrane reactors. The papers presented here demonstrate the potential of PMRs for a variety of applications, including water and wastewater treatment, air purification, and organic synthesis. However, there are still many challenges that need to be addressed to fully realize the potential of PMRs. These challenges include the development of new photocatalysts with improved performance, the optimization of reactor design, and the development of cost-effective and scalable PMR systems. Nonetheless, the research presented in this Special Issue provides a solid foundation for further developments in this exciting field.
